# Supplementary material for: Effect of Cooking and Domestic Storage on the Antioxidant Activity of Lenticchia di Castelluccio di Norcia, an Italian PGI Lentil Landrace
Source: Int J Environ Res Public Health. 2023 Jan 31;20(3):2585. doi: 10.3390/ijerph20032585 (PMC9916172; doi:10.3390/ijerph20032585)
Supplement: Supplementary file 1 [file ijerph-20-02585-s001.zip › ijerph-2075373-supplementary.pdf]

Effect of cooking and domestic storage on antioxidant activity of Lenticchia di Castelluccio di Norcia, an Italian PGI lentil landrace

Mattia Acito, Cristina Fatigoni, Milena Villarini and Massimo Moretti

Supplementary Figure

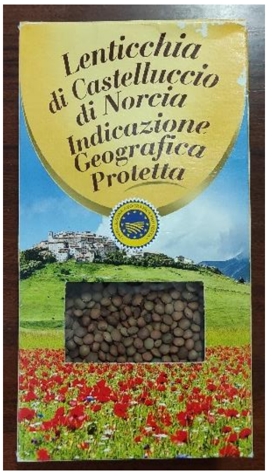

Figure S1. Package (250 g) of Lenticchia di Castelluccio di Norcia PGI.

Supplementary Tables

Table S1. Total Phenolic Content of Lenticchia di Castelluccio di Norcia PGI.

	Total Phenolic Content (mg GAE/g DM)	
**Cooking method**	**T_0**	**T_1**
None (raw)	9.080 ± 0.077 [bc]	7.759 ± 0.322 [bcd]
Boiled	4.937 ± 0.085 [ac]	3.924 ± 0.019 [ad]
Pressure-cooked	5.289 ± 0.039 [ab]	4.073 ± 0.102 [ad]

Results are expressed as mean ± standard error of the mean. Statistical significance: $p < 0.05$
[a] vs raw; [b] vs boiled; [c] vs pressure-cooked; [d] vs T_0

Table S2. Antioxidant activity of Lenticchia di Castelluccio di Norcia PGI assessed by DPPH assay.

	DPPH (μmol TE/g DM)	
**Cooking method**	**T_0**	**T_1**
None (raw)	33.019 ± 0.896 [bc]	29.227 ± 1.191 [bcd]
Boiled	8.420 ± 1.561 [ac]	8.782 ± 0.310 [ac]
Pressure-cooked	13.143 ± 0.161 [ab]	12.743 ± 1.071 [ab]

Results are expressed as mean ± standard error of the mean. Statistical significance: $p < 0.05$
[a] vs raw; [b] vs boiled; [c] vs pressure-cooked; [d] vs T_0

Table S3. Antioxidant activity of Lenticchia di Castelluccio di Norcia PGI assessed by ABTS assay.

	ABTS (μmol CE/g DM)	
**Cooking method**	**T_0**	**T_1**
None (raw)	32.122 ± 0.217 [bc]	31.772 ± 0.535 [bc]
Boiled	8.143 ± 0.212 [ac]	9.196 ± 0.695 [a]
Pressure-cooked	10.276 ± 0.719 [ab]	9.963 ± 0.719 [a]

Results are expressed as mean ± standard error of the mean. Statistical significance: $p < 0.05$
[a] vs raw; [b] vs boiled; [c] vs pressure-cooked

Table S4. Antioxidant activity of Lenticchia di Castelluccio di Norcia PGI assessed by ORAC assay.

	ORAC (μmol TE/g DM)	
**Cooking method**	**T_0**	**T_1**
None (raw)	3.580 ± 0.005 [b]	3.596 ± 0.005 [bc]
Boiled	3.540 ± 0.009 [a]	3.540 ± 0.013 [a]
Pressure-cooked	3.544 ± 0.015	3.523 ± 0.011 [a]

Results are expressed as mean ± standard error of the mean. Statistical significance: $p < 0.05$
[a] vs raw; [b] vs boiled; [c] vs pressure-cooked
